# Influence of *Citrus sunki* and *Poncirus trifoliata* Root Extracts on Metabolome of *Phytophthora parasitica*

**DOI:** 10.3390/metabo14040206

**Published:** 2024-04-05

**Authors:** Héros José Maximo, Francisca Diana da Silva Araújo, Carolina Clepf Pagotto, Leonardo Pires Boava, Ronaldo José Durigan Dalio, Gustavo Henrique Bueno Duarte, Marcos Nogueira Eberlin, Marcos Antonio Machado

**Affiliations:** 1 Biotechnology Laboratory, Centro de Citricultura Sylvio Moreira, Agronomic Institute, Cordeirópolis 13490-970, SP, Brazil; leonardo.boava@unar.edu.br (L.P.B.); rdalio@ideelab.com.br (R.J.D.D.);; 2 BioXyz Biotecnologia Microbiana e Bioprocessos e Industriais Ltda., Piracicaba 13414-224, SP, Brazil; 3 ThoMSon Mass Spectrometry Laboratory, Chemistry Institute, University of Campinas, UNICAMP, Campinas 13083-970, SP, Brazil; diana.araujo@ufpi.edu.br (F.D.d.S.A.); carolcpagotto@gmail.com (C.C.P.); gustavo_duarte95@hotmail.com (G.H.B.D.); marcos.eberlin@mackenzie.br (M.N.E.); 4 Campus Professora Cinobelina Elvas, Federal University of Piauí, Bom Jesus 64900-000, PI, Brazil; 5 Centro Universitário ‘Dr. Edmundo Ulson’—UNAR, Araras 13603-112, SP, Brazil; 6 School of Material Engineering and Nanotechnology, MackMass Laboratory, Mackenzie Presbyterian University, São Paulo 01302-907, SP, Brazil

**Keywords:** *Citrus sunki*, *Poncius trifloriata*, MALDI-MSI, UHPLC-ESI-Q-TOF, SCiLS, metabolomics

## Abstract

*Phytophthora parasitica* is an oomycete pathogen that infects a broad range of crops of worldwide economic interest; among them are citrus species. In general, some *Citrus* and the rootstocks of related genera offer considerable resistance against *P. parasitica*; therefore, understanding the mechanisms involved in the virulence of this pathogen is crucial. In this work, *P. parasitica* secondary metabolite production was studied using matrix-assisted laser desorption/ionization mass spectrometry imaging (MALDI-MSI) and ultrahigh-performance liquid chromatography coupled with electrospray ionization quadrupole time-of-flight tandem mass spectrometry (UHPLC/ESI-Q-TOF-MS) combined with chemometric tools, and its metabolic profile was evaluated under the influence of *Citrus sunki* (a highly susceptible host) and *Poncirus trifoliata* (a resistant genotype) extracts. The root extracts of *Citrus sunki* had an influence on the growth and hyphae morphology, and the root extracts of *P. trifoliata* had an influence on the zoospore behavior. In parallel, the spatial distribution of several metabolites was revealed in *P. parasitica* colonies using MALDI-MSI, and the metabolite ion of *m/z* 246 was identified as the protonated molecule of Arg-Ala. The MALDI-MSI showed variations in the surface metabolite profile of *P. parasitica* under the influence of the *P. trifoliata* extract. The *P. parasitica* metabolome analysis using UHPLC-ESI-Q-TOF-MS resulted in the detection of Arg-Gln (*m*/*z* 303.1775), as well as L-arginine (*m*/*z* 175.1191) and other unidentified metabolites. Significant variations in this metabolome were detected under the influence of the plant extracts when evaluated using UHPLC-ESI-Q-TOF-MS. Both techniques proved to be complementary, offering valuable insights at the molecular level when used to assess the impact of the plant extracts on microbial physiology in vitro. The metabolites identified in this study may play significant roles in the interaction or virulence of *P. parasitica*, but their functional characterization remains to be analyzed. Overall, these data confirm our initial hypotheses, demonstrating that *P. parasitica* has the capabilities of (i) recognizing host signals and altering its reproductive programing and (ii) distinguishing between hosts with varying responses in terms of reproduction and the production of secondary metabolites.

## 1. Introduction

Terrestrial plants and microorganisms, particularly oomycetes, have been in coevolution for more than 320 million years [1]. Over this period, a sophisticated biochemical communication system has evolved, facilitating both mutualistic and parasitic interactions. In plant–pathogen interactions, generally, the pathogens possess an arsenal of secreted effector molecules that interact with plant receptors/proteins to manipulate plant physiology and immunity [2]. On the other hand, plants have developed molecular antennae to detect/recognize pathogen-associated molecular patterns and/or effectors in order to activate their defense [3]. The key factors of this complex biochemical warfare are still obscure for most pathogens and their host plants [4].

Adapted parasitic microorganisms are able to respond to shifts in pH, temperature, compound composition, and other abiotic stresses in the environment [5]. The oomycete *Phytophthora parasitica* Dastur (syn. *Phytophthora nicotianae* Breda van Haan) is a soil-borne filamentous plant pathogen that resembles a fungus and is able to infect more than 250 plant families worldwide [6]. It is associated with plant diseases in tropical and subtropical areas and is known for its resilience and adaptability. *P. parasitica* may recognize environmental shifts related to either (i) abiotic stresses, responding by producing chlamydospores, for example (a reproductive structure related to stress), or (ii) recognizing “hosts signals” or root exudates, responding through active growth and the release of zoospores that can swim towards a biochemical gradient from the plant to start an infection [7]. It is believed that when a pathogen detects the presence of a nearby host, it is highly probable that it not only alters its growth and zoospore activity but also undergoes changes in its physiology and metabolism. This can lead to the production of various secondary metabolites that play a crucial role in the initial stages of the interaction with the host.

*P. parasitica* is one of the main citrus pathogens that causes damage and economic losses in citrus orchards and nurseries worldwide [6]. This pathogen can infect all plant organs and tissues, compromising productivity and leading to mortality. The main diseases caused by *P. parasitica* in citrus are citrus gummosis and citrus root rot. Until now, there have not been any practical, sustainable control methods available for these diseases, and the usual approach is to remove and destroy the infected plants from orchards or nurseries [8]. Different scion and rootstock varieties present distinct levels of resistance and susceptibility to the diseases. For instance, the rootstocks *Poncirus trifoliata* and *Citrus sunki* show opposite outcomes when infected: the former is highly tolerant, while the latter is frequently very susceptible [9]. However, it is not known what causes these dramatic differences in their relationship with *P. parasitica*. Thus, it is evident that there is a need for more studies to decipher the main molecular factors driving the susceptibility and resistance in the *P. parasitica*–citrus interaction.

Metabolomics is a research approach designed to uncover the small molecules generated by a biological system, taking into account factors such as the composition, the interactions, and how the system responds to changes in its environment [10,11]. Mass spectrometry (MS) techniques have been widely applied in this area [12]. Matrix-assisted laser desorption/ionization mass spectrometry imaging (MALDI-MSI) has also been applied in metabolomics studies, offering the opportunity to monitor the spatial and temporal distributions of metabolites [13,14,15]. Recently, MALDI-MSI was successful in revealing secondary metabolites involved in intracellular metabolism [16], as well as monitoring metabolic exchanges in interspecies relationships [17,18]. Also, ultrahigh-performance liquid chromatography coupled with electrospray ionization quadrupole time-of-flight tandem mass spectrometry (UHPLC-ESI-Q-TOF MS) has been widely used in metabolomic studies since it offers prior and high-efficiency separation followed by fast and selective detection using MS [19,20,21,22]. The chromatography separates metabolites over time, reducing ion suppression during ionization, producing cleaner spectra, and therefore providing a broader coverage of metabolites [12].

In this work, we showed for the first time the use of MALDI-MSI and UHPLC-ESI-Q-TOF MS to study the secondary metabolism of *P. parasitica* monocultures and *P. parasitica* under the influence of *Citrus sunki* (a highly susceptible host) and *P. trifloriata* (a resistant genotype) extracts. Our main scientific questions were as follows: (i) Is *P. parasitica* able to recognize plant signals in the environment and actively change its growth, zoospore behavior, and secondary metabolism to enable infection? (ii) Does *P. parasitica* recognize different host signals (from a resistant and a susceptible host) and change its growth, zoospore behavior, and secondary metabolism accordingly? We hypothesized that *P. parasitica* would respond to signals from plants by altering its growth, zoospore behavior, and secondary metabolism and that this would occur in a different fashion for each different host. Our goals for this study were to address these questions, assess the effectiveness of utilizing MALDI-MSI and UHPLC-ESI-Q-TOF MS techniques, and select target metabolites for further functional characterization.

## 2. Materials and Methods

Strains and materials: *P. parasitica* isolate IAC 01/95 was maintained in solid culture medium M1. The conductive indium tin oxide (ITO)-coated glass slides and peptide calibration standard II were purchased from Bruker Daltonics (Bremen, Germany), and the a-cyano-4-hydroxy-cinnamic acid (CHCA, 99%), trifluoroacetic acid (TFA, 99%), acetonitrile (≥99.9%), and methanol (≥99.9%) HPLC grade from Sigma-Aldrich (Steinheim, Germany).

Growth rate, hyphae morphology, sporangia development, and zoospore behavior: Effect of extracts on growth: *P. parasitica* isolate IAC 01/95.1 was transferred to the center of a Petri dish containing M1 medium (L-Asparagin 0.3 g L^−1^, FeSO_4_·7 H_2_O 1.5 mg L^−1^, CaCl_2_·2 H_2_O 15.0 mg L^−1^, MgSO_4_·7 H_2_O 0.15 g L^−1^, KH_2_PO_4_ 0.705 g L^−1^, K_2_HPO_4_ 0.39 g L^−1^, Thiaminhydrochlorid 1.5 mg L^−1^, ZnSO_4_·7 H_2_O 1.5 mg L^−1^, CuSO_4_·5 H_2_O 0.03 mg L^−1^, Na_2_MoO_4_·4 H_2_O 0.03 mg L^−1^, MnCl_2_·4 H_2_O 0.03 mg L^−1^, Glucose 3 g L^−1^, Agar 18 g L^−1^) and incubated at 25 °C in the dark. After one week, *P. parasitica* was again transferred to M1-medium Petri dishes supplemented, or not, with *Citrus sunki* (a susceptible variety) or *Poncirus trifoliata* (a tolerant variety) root extracts. The root extracts were produced by grinding 1 g of roots in liquid nitrogen. The homogenized solution was solubilized in 10 mL of phosphate buffer, pH 7.2, and supplemented with 90 mL of M1 medium. The control treatment had the M1 medium supplemented with 10 mL of phosphate buffer. We monitored mycelial growth on a daily basis by measuring the colony radius. This observation was carried out for a total of five days using six Petri dishes for each treatment (*n* = 6) with two biological replicates.

Effect of extracts on the hypha branch: in order to verify the morphology and branching of *P. parasitica* hyphae in all treatments, we used an optical microscope. The observations were undertaken daily for five days. Effect of extract on sporangia development and zoospore behavior: after the colony reached up to 80% of M1-containing plates supplemented with *C. sunki* and *P. trifoliata* root extract, the development of sporangia was induced by pouring sterile water into the plaque and replacing it daily for one week. To trigger the release of zoospores, the plates were kept in the dark at 4° C for one hour. The quantity of the released zoospores was then measured using a Neubauer chamber. To verify the motility of the zoospores in the presence of extracts of citrus roots and water, we used a concave microscopy glass filled with water or root extracts and analyzed it under an optical microscope. The germination of encysted zoospores was performed by pipetting 1 × 10^4^ encysted zoospores in dialysis membranes (1 cm^2^) soaked with 1 mL of water or root extracts of *C. sunki* and *P. trifoliata*. We performed Student’s T-test to compare the means of growth rate and the number of zoospores released.

Sample preparation for MALDI-TOF MSI: *P. parasitica* was grown in M1 medium, remaining in B.O.D. at 25 °C. Subsequently, a small section of the agar, containing the oomycete cells, was cut with a stylet and inoculated on MALDI glass slides. These slides had been previously placed within Petri plates (100 × 20 mm) with a thin layer of each medium (M1 agar medium; M1 agar medium plus *C. sunki* extract; M1 agar medium plus *P. trifloriata* extract). The cultures were inoculated for 36 h at 25 °C; then, the slides were removed from the Petri plate and dehydrated in a desiccator under vacuum. The matrix consisted of a saturated CHCA solution prepared using acetonitrile–water (1:1) with 2.5% TFA and was manually sprayed on the slides. A photography of the slides was taken before and after the matrix application.

MALDI-MSI analysis The slides were supported in an MTP slide-adapter II (Bruker Daltonics, Bremen, Germany) and loaded into the Autoflex III Smartbeam Bruker Daltonics MALDI-TOF spectrometer. The instrument was operated in positive ion reflector mode, with a mass range of *m*/*z* 100–1500 with 600 laser shots, and the laser power was adjusted to 50%. The ion source 1, the ion source 2, the lens, the reflector, and the reflector 2 voltages were, respectively, set to 20.00, 17.47, 8.80, 22.00, and 10.10 kV. The pulsed ion extraction time was 30 ns, the suppression mass gate was set to *m*/*z* 100, and the detector gain reflector was 7.0×. The calibration of the equipment was performed by using peptide calibration standard II and matrix ions. The processing method was FC_PepMix, which corresponds to a parameter set for picking, smoothing, and subtracting the database. All MSI experiments were performed in duplicates.

Data analysis using FlexImaging: The spectra were internally calibrated by matrix ions and the datasets were analyzed using FlexImaging 4.0 (Bruker Daltonics, Bremen, Germany), in which the ion of interest was manually filtered from the mean or individual spectra using 0.5–1.0 Da increments. The images were normalized using TIC mode, and a hypothetical color was assigned to each specific ion [15,17,23].

Data analysis using SCiLS Lab: The image datasets were analyzed using the software SCiLS Lab (SCiLS GmbH, Bremen, Germany). The dataset with a minimal *m*/*z* value of 150–650 Da and an interval width of ±0.2 Da was used and submitted to baseline removal, normalization regarding the total ion count (TIC), and peak alignment. The three datasets (*Pp*, *Pp*/*Cs*, and *Pp*/*Pt*) were compared using principal component analysis (PCA) and probabilistic latent semantic analysis (pLSA) applied to the individual and mean spectra.

*P. parasitica* extract preparations for UHPLC-ESI-Q-TOF analysis: *P. parasitica* was separately inoculated on M1 agar medium, M1 agar medium plus *C. sunki* extract, and M1 agar medium plus *P. trifloriata* extract, and submitted to incubation in B.O.D. at 25 °C for 36 h. Small portions of the agar containing the cells were transferred to Eppendorf tubes. Methanol–water (1:1, 500 µL) was added to each sample, which were sonicated for 20 min and centrifugated for 10 min. The cells were separated, and the supernatants were diluted and analyzed using UHPLC-ESI-Q-TOF-MS.

UHPLC-ESI-Q-TOF-MS analysis: The extracts were analyzed in a 6550 iFunnel Q-TOF LC/MS mass spectrometer (Agilent Technologies) using a Titan C18 column (10 cm × 2.1 mm, 1.9 µm) from Supelco. The injection volume was 5 mL, and the column temperature was maintained at 40 °C. The flow rate was 0.4 mL min^−1^ developed in a gradient program ranging from 5 to 100% of solvent B (solvent A = water containing 0.1% of formic acid; solvent B = acetonitrile). The capillary voltage was set at 3000 V, the nozzle voltage at 320 V, the gas temperature at 290 °C, the pressure of nebulizer gas at 45 psig, the sheath gas at 12 L min^−1^ and 350 °C, and the Fragmentor and Octopole 1RF Vpp voltages were set at 100 V and 750 V, respectively. The analyses were made in the scan range of *m*/*z* 100–1500.

Data processing and chemometric analysis: The spectral mass datasets were obtained using the Mass Hunter Qualitative software (Agilent Technologies, version B.07.00). These data were exported to open-source software XCMS [24,25] for peak alignment and, after, analyzed using Metaboanalyst 3.0 [26] for chemometric analysis PCA.

## 3. Results and Discussion

### 3.1. Growth Rate, Hyphae Morphology, Sporangia Development, and Zoospore Behavior

**Figure 1:**
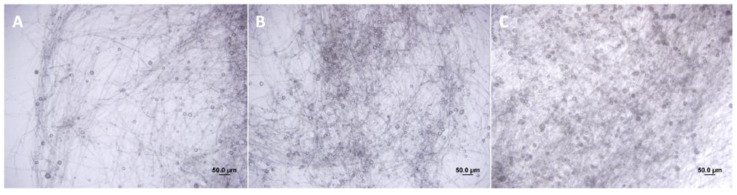
Sporangia development of *P. parasitica* untreated (**A**), treated with *C. sunki* (**B**), and *P. trifoliata* (**C**) root extracts.

According to our hypothesis, the root extracts of C. sunki and P. trifoliata altered the growth rate of *P. parasitica* hyphae (Figure 1). After treatment with *C. sunki* extracts, *P. parasitica* showed an advancement in sporangia development in one day and exhibited a higher quantity of sporangia compared to treatment with root extracts of *P. trifoliata* and control treatment (Figure 1). The number of zoospores released was significantly higher after treatment with root extracts of *C. sunki*, i.e., 6 × 10^5^ per mL, when compared to root extracts of *P. trifoliata* and control treatment, i.e., 8 × 10^4^ and 1 × 10^4^ per mL, respectively. The encystment rate and motility of the zoospores after treatment with root extracts of *C. sunki* were not altered (Table 1). On the other hand, the encystment rate and motility of the zoospores were altered after treatment with root extracts of *P. trifoliata*.

**Figure 2:**
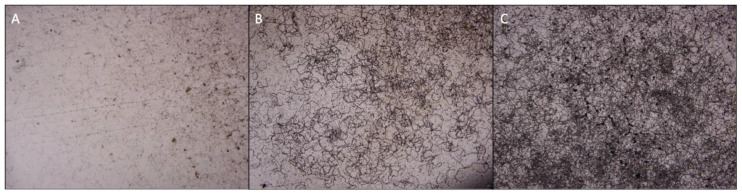
Germination of encysted zoospores of *P. parasitica* in dialysis membranes soaked with water (**A**), *P. trifoliata* (**B**), and *C. sunki* (**C**) root extracts.

The initial stage of plant infection by oomycetes relies on the ability of biflagellated zoospores to reach host tissues. In the rhizosphere environment, the plants emit attractive chemical signals, which direct the zoospores of the pathogen to the sites of infection [27,28]. In this study, it is worth noticing that the encystment rate and the motility of zoospores in *P. parasitica* were significantly impacted over time by the treatment with *P. trifoliata* root extracts. Zoospores started to spin before encysting (Table 1). Since *P. parasitica* zoospores present two flagella that give it the capability to swim towards a gradient, the spinning behavior could be explained by the motility of only one flagellum before encystment. The increase both in spinning and in the encystment rate of zoospores is generally related to environmental stresses [7]. So, it is likely that *P. trifoliata* root extracts contain certain toxic compounds that hinder zoospore activity, thereby reducing the size of the inoculum during the initial stages of infection. On the other hand, the susceptible *C. sunki* plants presented root extracts that did not affect the zoospores’ motility or encystment, suggesting that there is no toxic effect, enabling zoopores’ proximity and infection. Furthermore, the germination of encysted zoospores was also altered by both extracts, with a more pronounced effect observed in *P. parasitica* after exposure to *C. sunki* extracts (Figure 2).

Overall, the data indicate that the *C. sunki* extracts promote infection, whereas *P. trifoliata* extracts compromise it. This suggests that the dynamics of host and non-host/resistance relations between plants and *P. parasitica* begin before the actual infection.

### 3.2. MALDI-MSI 

**Figure 3:**
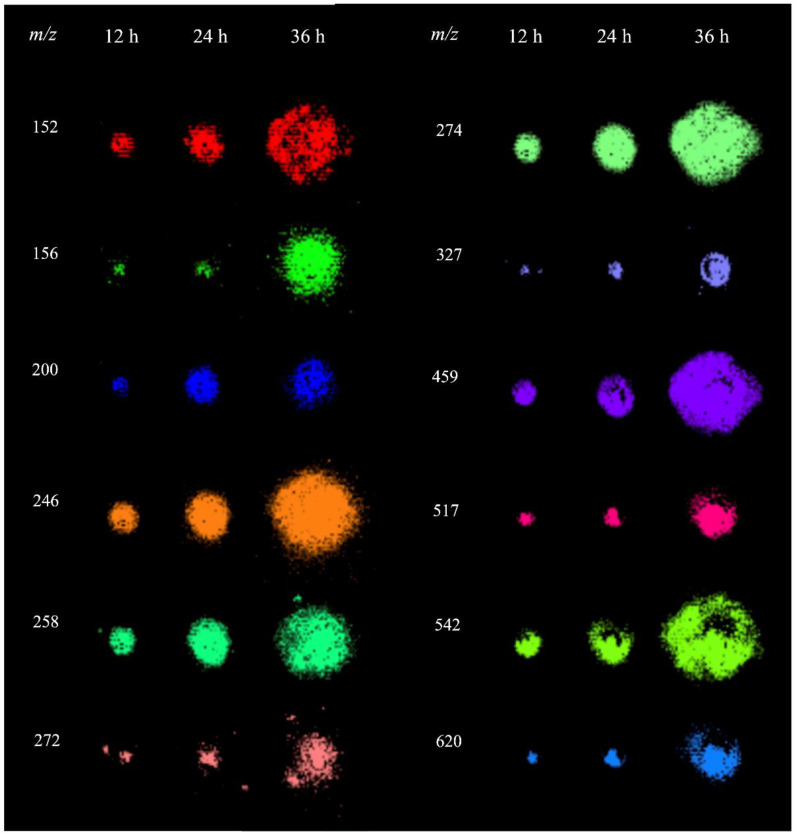
MALDI-MSI of metabolites detected in *P. parasitica*.

The analysis of *P. parasitica* colonies using MALDI-MSI revealed several metabolites in the *m*/*z* range of 150–650, for which the spatial and temporal distributions are shown in Figure 3. To characterize these ions, extracts from *P. parasitica* were analyzed using electrospray ionization coupled with Fourier transform ion cyclotron resonance mass spectrometry (ESI-FT-ICR-MS) and MALDI-MS/MS. This allowed for the precise measurement of *m*/*z* with high accuracies and allowed us to obtain their fragmentation patterns (Table S1). These data were used for searches in the MassBank, METLIN, and LIPID MAPS databases [29,30,31]. The ion of *m*/*z* 246.1558 with a molecular formula C_9_H_19_N_5_O_3_ was identified as the protonated molecule of Arg-Ala.

**Figure 4:**
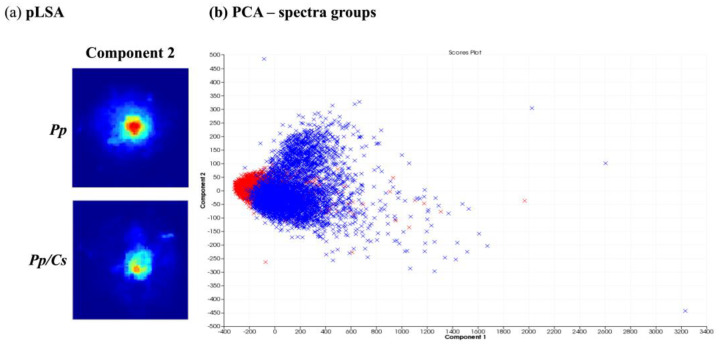
Comparison between *P. parasitica* grown on M1 agar medium (*Pp*) and on M1 agar medium plus *C. sunki* extract (*Pp*/*Cs*): (**a**) pLSA of eight components, in which only components with clearly visible structures are shown; (**b**) PCA of individual spectra of each image obtained (*Pp*—blue; *Pp/Cs*—red).

After obtaining spatial and temporal data on the distribution of several ions originating from metabolites produced by *P. parasitica* monocultures using MALDI-MSI, its metabolic production was evaluated under the influence of *C. sunki* and *P. trifloriata* extracts. For this, the plant extracts were added to the M1 agar medium. The MSI obtained were compared and evaluated using the SCiLS Lab software (SciLS GmbH). Figure 4 shows the results of the chemometric analysis when comparing the images of *P. parasitica* on M1 agar medium (*Pp*) and on M1 agar medium plus *C. sunki* extract (*Pp*/*Cs*). The pLSA analysis revealed that the same component was described in both samples (Figure 4a), therefore suggesting that there are no significant differences between the *Pp* and *Pp*/*Cs* samples. PCA (Figure 4b) shows that individual spectra are almost fully overlapping. The MALDI-MSI suggested, therefore, that *P. parasitica* keeps its surface metabolite profile under the influence of *C. sunki* extract.

**Figure 5:**
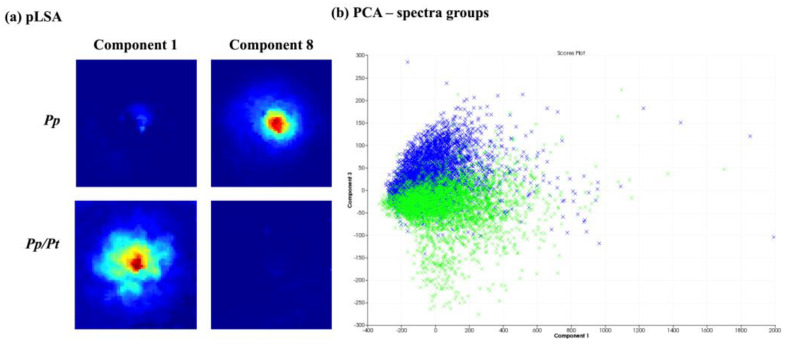
Comparison between *P. parasitica* grown on M1 medium (*Pp*) and on M1 medium plus *P. trifluoriata* extract (*Pp*/*Pt*). (**a**) pLSA of 10 components, in which only components with clearly visible structures are showed. (**b**) PCA of the individual spectra of each image obtained (*Pp*—blue; *Pp*/*Pt*—green).

Figure 5 presents the chemometric analysis of the image datasets of *P. parasitica* on M1 agar medium (*Pp*) and on M1 agar medium plus *P. trifloriata* extract (*Pp*/*Pt*). The pLSA showed that two components separate the two samples (Figure 5a). The PCA (Figure 5b) was also able to partially discriminate the samples via PC3. Therefore, the MALDI-MSI shows that the surface metabolite profile of *P. parasitica* seems to be altered under the influence of *P. trifoliata* extracts.

**Figure 6:**
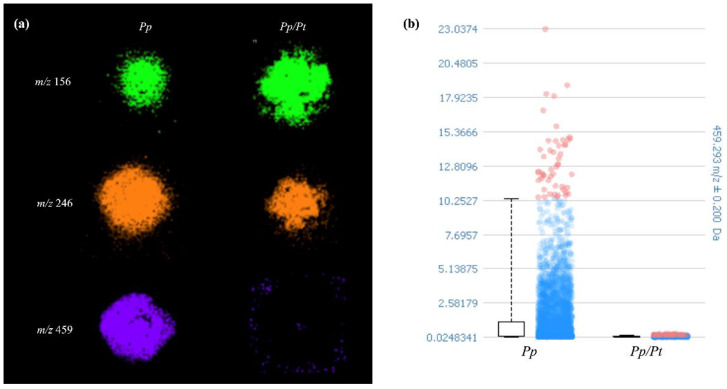
(**a**) Spatial metabolite distributions that varied in *P. parasitica* (*Pp*) under the influence of *P. trifloriata* extract (*Pp/Pt*). (**b**) Box plot of the signal intensity of metabolite of *m*/*z* 459.

To find the discriminating ions of the image datasets of *Pp* and *Pp/Pt* samples, the receiver operating characteristic (ROC) tool was used. The area under the ROC curve (AUC), presenting values between 0 and 1, expresses the discrimination power of the *m*/*z* value, meaning that when these values are closer to 0 or 1, the ion is predominantly present in a sample [27]. The ion of *m*/*z* 246, identified as protonated Arg-Ala, showed an AUC value of 0.497, which means that its production remained nearly constant when the oomycete was under the influence of *P. trifloriata* extract. However, the ions of *m*/*z* 156 with and AUC value of 0.776 and those of *m*/*z* 459 with an AUC value of 0.298, previously detected in *P. parasitica*, showed significantly different relative abundances in the *Pp* or *Pp*/*Pt* samples (Figure 6). In general, dipeptides such as Arg-Ala are generated through the degradation of proteins or polypeptides as sources of amino acids for various cellular processes. However, as far as we know, there is no description of a specific role for these peptides in plant–pathogen interactions.

### 3.3. UHPLC-ESI-Q-TOF-MS

**Figure 7:**
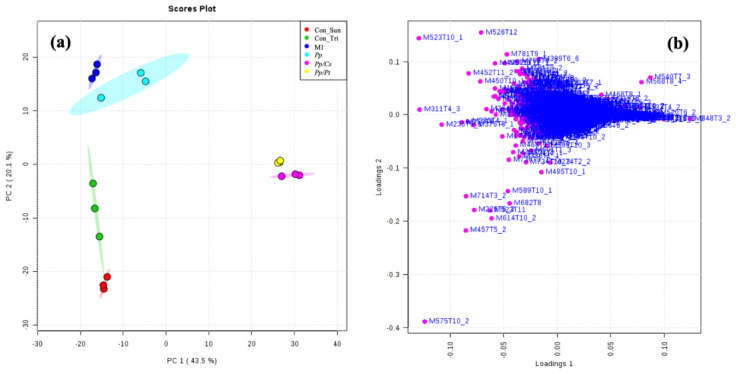
PCA: (**a**) score and (**b**) loading plots. Con_M1: M1 agar medium extract (control); *Pp*: *P. parasitica* extract; Con_Tri: M1 agar medium plus *P. trifloriata* extract; *Pp*/*Pt*: *P. parasitica* grown on M1 agar medium plus *P. trifloriata* extract; Con_Sun: M1 agar medium plus *C. sunki* extract; *Pt*/*Cs*: *P. parasitica* grown on M1 agar medium plus *C. sunki* extract.

To compare the metabolomes of *P. parasitica* on M1 agar medium and under the influence of *C. sunki* and *P. trifloriata*, extracts of the oomycete were prepared in these three different conditions with an incubation time of 36 h and then analyzed using UHPLC-ESI-Q-TOF-MS. The datasets were submitted to the chemometric analysis using the online software XCMS and MetaboAnalyst. PCA, indeed, resulted in isolated clusters for all samples (Figure 7a). The *Pp* samples were placed next to the Con_M1 samples (control of M1 agar medium), demonstrating that the oomycete produces few additional metabolites in an incubation time of 36 h. The specific metabolites produced by *P. parasitica* were selected according to the loading plot (Figure 7b,d and Figure S1) (see Table 2). In an attempt to identify these metabolites, the high-accuracy m/z values were measured and used together with the fragmentation patterns to search the METLIN, Massbank, and LIPIS MAPS databases [23,24,25]. The ions of *m*/*z* 175 and 303 were, respectively, identified as protonated L-arginine and Arg-Gln.

**Figure 8:**
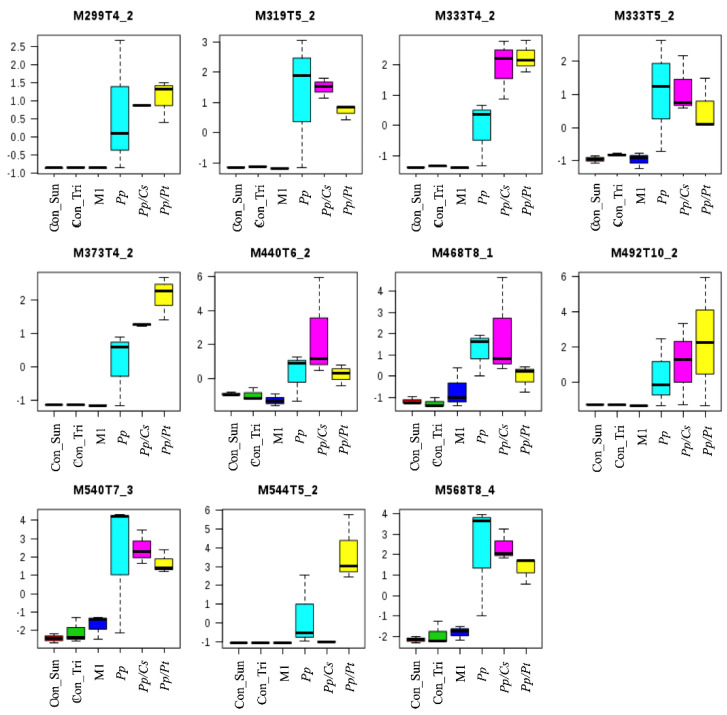
Metabolites of *P. parasitica* (*Pp*) that varied under the influence of *P. trifloriata* (*Pp*/*Pt*) and *C. sunki* (*Pp*/*Cs*) extracts. Con_M1: M1 agar medium extract (control); Con_Tri: M1 agar medium plus *P. trifloriata* extract; Con_Sun: M1 agar medium plus *C. sunki* extract.

PCA showed significant separations of the *Pp* sample from both the *Pp*/*Cs* and *Pp*/*Pt* samples, suggesting that the metabolic profile of *P. parasitica* is indeed substantially altered under the influence of these plant extracts. Table S1 and Figure S2 show the discriminant ions observed in the *Pp*/*Cs* and *Pp*/*Pt* sample regions of the loading plot. The ions of *m*/*z* 299, 319 (T5_2), 333 (T4_2 and T5_2), 373, 440, 468, 492, 540, and 568 were detected in the three samples (*Pp*, *Pp/Cs*, and *Pp/Pt*), and that of *m*/*z* 544 was common only in the *Pp* and *Pp/Pt* samples (Figure 8). The relative abundance of these ions varied significantly under the influence of the plant extracts. For instance, the ion of *m*/*z* 333 (T4_2), 373, and 492 increased under the influence of both extracts, whereas the ion of *m*/*z* 544 only increased under the influence of *P. trifoliata* extract and failed to be detected in the *Pp/Cs* sample.

Interestingly, the ion of *m*/*z* 649 was exclusive to the *Pp*/*Cs* sample, whereas the ions of *m*/*z* 190, 303 (T6_2), 330 (T3_4), and 520 were exclusive to the *Pp*/*Pt* sample (Figures S2 and S3), which seem to be produced by oomycetes only under these specific conditions. The metabolite ions of *m*/*z* 179, 228, 249, 254, 280, 297, 311, 319 (T3, T4_3, and T6_1), 330 (T5_5), 335 (T3_2, T4_4, and T5_3), 348, 370, 372, 377 (T4_3, and T5_2), 385, 395, 400, 402, 418, 420, 439, 474, 479, 523, 546 (T8_3, and T9_3), 564, 570, 604 (T8_4, and T9), 605, 619, 633, and 665 were detected in both *Pp*/*Cs* and *Pp*/*Pt* samples, which, therefore, are probably additional metabolites produced by *P. parasitica* under the influence of the two plant extracts (Figures S2 and S3). Through analyzing the plant extracts (Figure S4), it was observed that the ions of *m*/*z* 212, 245 (T3_2), 252, 490 (T2_1), 520, 589, 734, 765 (T11_1), 765 (T11_3), and 787 were exclusive to the *C. sunki* extract and that they may be correlated to the susceptibility of this plant to *P. parasitica*. These ions are targets for functional characterization.

## 4. Conclusions

Overall, these data confirm our initial hypotheses, demonstrating that *P. parasitica* has the capabilities of (i) recognizing host signals and altering its reproductive programing and (ii) distinguishing between hosts with varying responses in terms of reproduction and the production of secondary metabolites. The combined use of MALDI-MSI and UHPLC-ESI-Q-TOF-MS provided a comprehensive perspective on the spatial and temporal metabolome of *P. parasitica*. An amino acid and two dipeptides were identified, and several other key unknown metabolites were detected under the influence of the root extracts. The MS data, when combined to the chemometric analysis, were able to compare metabolites and to show their increments and suppressions, thus generating complementary results for the in vitro evaluation of *P. parasitica* metabolome under the influence of plant extracts. The *P. trifoliata* root extracts showed a toxic effect on the zoospores of *P. parasitica* and changed its metabolome in general, suggesting that it may present some inhibitory or toxic effect to the pathogen physiology. Along with another unknown features related to the defense system of this plant, this result may help explain its resistance against the pathogen. The *C. sunki* extract significantly stimulated the reproduction of *P. parasitica* and did not exhibit any inhibitory effect on zoospores. In the following treatment with *C. sunki*, certain unidentified secondary metabolites were activated, making them potential targets for identification and functional characterization. These metabolites may play a role in the success of the infection or the virulence of the pathogen.

## Figures and Tables

**Table 1:** Encystment rate and zoospore mobility of *P. parasitica* treated with water or *C. sunki* or *P. trifoliata* root extracts.

	*Sunki*		*Trifoliata*	
	Encystment Rate	Zoospores Mobility	Encystment Rate	Zoospores Mobility
Water	None	Active	None	Active
Root extract	None	Active	Increased	Spinning

**Table 2:** Metabolites of *P. parasitica* detected using UHPLC-ESI-Q-TOF-MS.

*m*/*z*	VIP Scores	*p* Value	Retention Time (Min)	MS/MS Fragment Masses	Type of Ion	Putative Assignments	D (ppm)
175.1191	0.85	0.008	0.37	175, 158, 130, 116, 70, 60	[M + H]^+^	L-Arginine	1
296.1431	0.56	0.020	5.65	-	-	-	No match
303.1775	0.48	0.011	0.42	303, 286, 175, 130, 112, 70, 60	[M + H]^+^	Arg-Gln	0
303.2320	1.66	0.011	6.81	303, 233, 208, 191, 175, 167, 135, 118, 102, 93, 71	-	-	No match
313.2722	0.42	0.083	5.00	-	-	-	No match
392.3133	0.24	0.034	7.08	392, 371, 336, 311, 276, 242, 224, 194, 182, 130, 118, 102, 89, 59	-	-	No match
399.1792	1.03	0.020	5.69	-	-	-	No match
401.2298	1.02	0.011	4.70	401, 340, 264, 194, 181, 158, 131, 118, 101	-	-	No match
489.2270	0.85	0.015	6.78	489, 240, 119, 105, 91	-	-	No match
721.6454	0.08	0.018	9.03	-	-	-	No match

## Data Availability

The raw data supporting the conclusions of this article will be made available by the authors on request.

## Supplementary Materials

The following supporting information can be downloaded at: https://www.mdpi.com/article/10.3390/metabo14040206/s1, Figure S1: Box plot of the signal intensity of P. parasitica metabolites detected by UHPLCESI-Q-TOF-MS and selected by PLS-DA loading plot; Figure S2: Discriminant metabolites of *Pp/Pt* and *Pp/Cs* samples; Figure S3: Discriminant metabolites of *Pp/Pt* and *Pp/Cs* samples (continuation); Figure S4: Discriminant metabolites of Con_Pt and Con_Cs samples. Sun = *Pt/Cs*; Tri = *Pp/Pt*; Table S1: Specific metabolites of P. parasitica detected by MALDI-MSI; Scheme S1. Steps for microbial MALDI-MSI developed in this work.
