# Supplementary material for: Influence of Citrus sunki and Poncirus trifoliata Root Extracts on Metabolome of Phytophthora parasitica
Source: Metabolites. 2024 Apr 5;14(4):206. doi: 10.3390/metabo14040206 (PMC11052222; doi:10.3390/metabo14040206)
Supplement: Supplementary file 1 [file metabolites-14-00206-s001.zip › metabolites-2737787-supplementary.pdf]

Supporting information

Influence of *Citrus sunki* and *Poncirus trifoliata* Root Extracts on Metabolome of *Phytophthora parasitica*

Héros José Maximo [1,2,*], Francisca Diana da Silva Araújo [3,4], Carolina Clepf Pagotto [3], Leonardo Pires Boava [1,5], Ronaldo José Durigan Dalio [1], Gustavo Henrique Bueno Duarte [3], Marcos Nogueira Eberlin [3,6] and Marcos Antonio Machado [1]

[1] Biotechnology Laboratory, Centro de Citricultura Sylvio Moreira, Agronomic Institute,
Cordeirópolis, SP 13490-970, Brazil; leonardo.boava@unar.edu.br (L.P.B.); rdalio@ideelab.com.br (R.J.D.D.); marcos@ccsm.br (M.A.M.)

[2] BioXyz Biotecnologia Microbiana e Bioprocessos e Industriais Ltda., Piracicaba, SP 13414-224, Brazil

[3] ThoMSon Mass Spectrometry Laboratory, Chemistry Institute, University of Campinas, UNICAMP,
Campinas, SP 13083-970, Brazil; diana.araujo@ufpi.edu.br (F.D.d.S.A.); carolcpagotto@gmail.com (C.C.P.);
gustavo_duarte95@hotmail.com (G.H.B.D.); marcos.eberlin@mackenzie.br (M.N.E.)

[4] Campus Professora Cinobelina Elvas, Federal University of Piauí, Bom Jesus, PI 64900-000, Brazil

[5] Centro Universitário 'Dr. Edmundo Ulson'—UNAR, Araras, SP 13603-112, Brazil

[6] School of Material Engineering and Nanotechnology, MackMass Laboratory, Mackenzie Presbyterian
University, São Paulo, SP 01302-907, Brazil

* Correspondence: heros@bioxyz.com.br; Tel.: +55-19-999829900

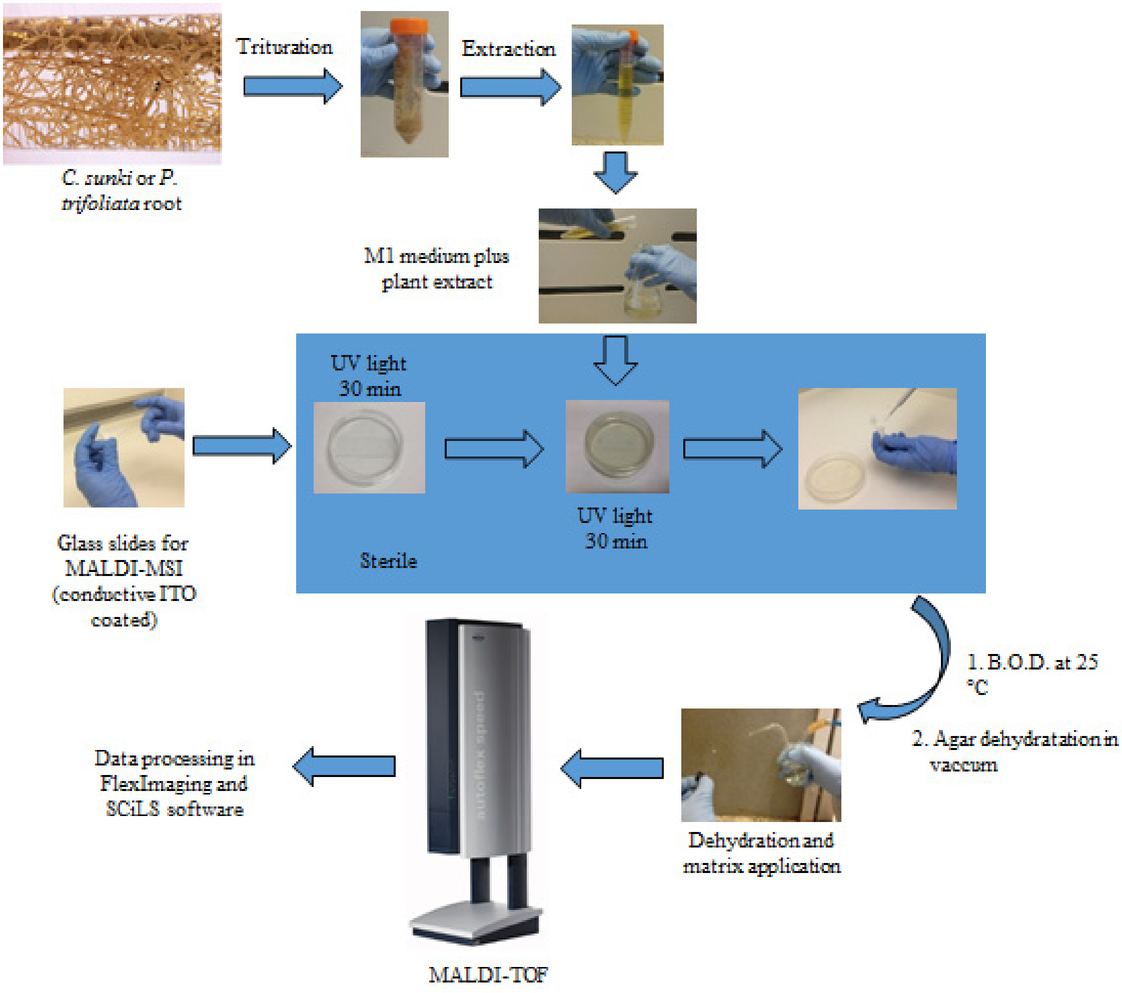

Scheme S-1. Steps for microbial MALDI-MSI developed in this work.

Table S-1. Specific metabolites of *P. parasitica* detected by MALDI-MSI.

MALDI-MSI	ESI-FT-ICR-MS	MS/MS fragment masses	Type of ion	Molecular formula	Putative assignments	Δ (ppm)
152	152.0941	-	-	-	No match	-
156	156.1210	156, 117, 104, 70, 60	-	-	No match	-
200	200.1724	-	-	-	No match	-
246	246.1558	246, 229, 212, 132, 112, 70, 60	$[M + H]^+$	$C_9H_{19}N_5O_3$	Arg-Ala	1
258	258.1737	-	-	-	No match	-
459	459.3078	458, 441, 315, 291, 165, 133, 89	-	-	No match	-
468	468.3891	468, 400, 373, 317, 285, 174, 114, 58	-	-	No match	-
517	517.3705	-	-	-	No match	-
542	542.2656	541, 509, 461, 355, 300, 121	-	-	No match	-
620	620.6166	-	-	-	No match	-

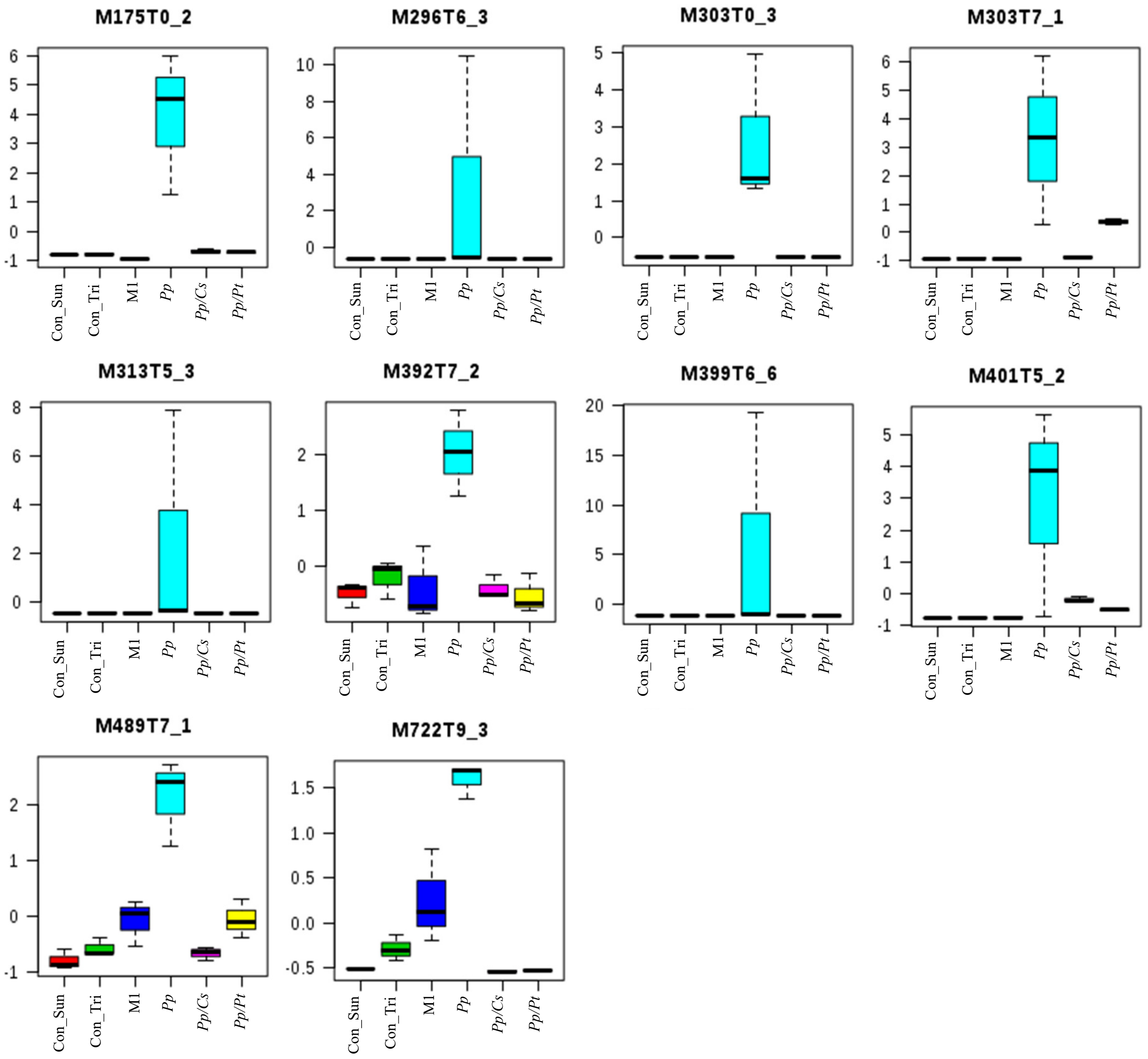

Figure S-1. Box plot of the signal intensity of *P. parasitica* metabolites detected by UHPLC-ESI-Q-TOF-MS and selected by PLS-DA loading plot.

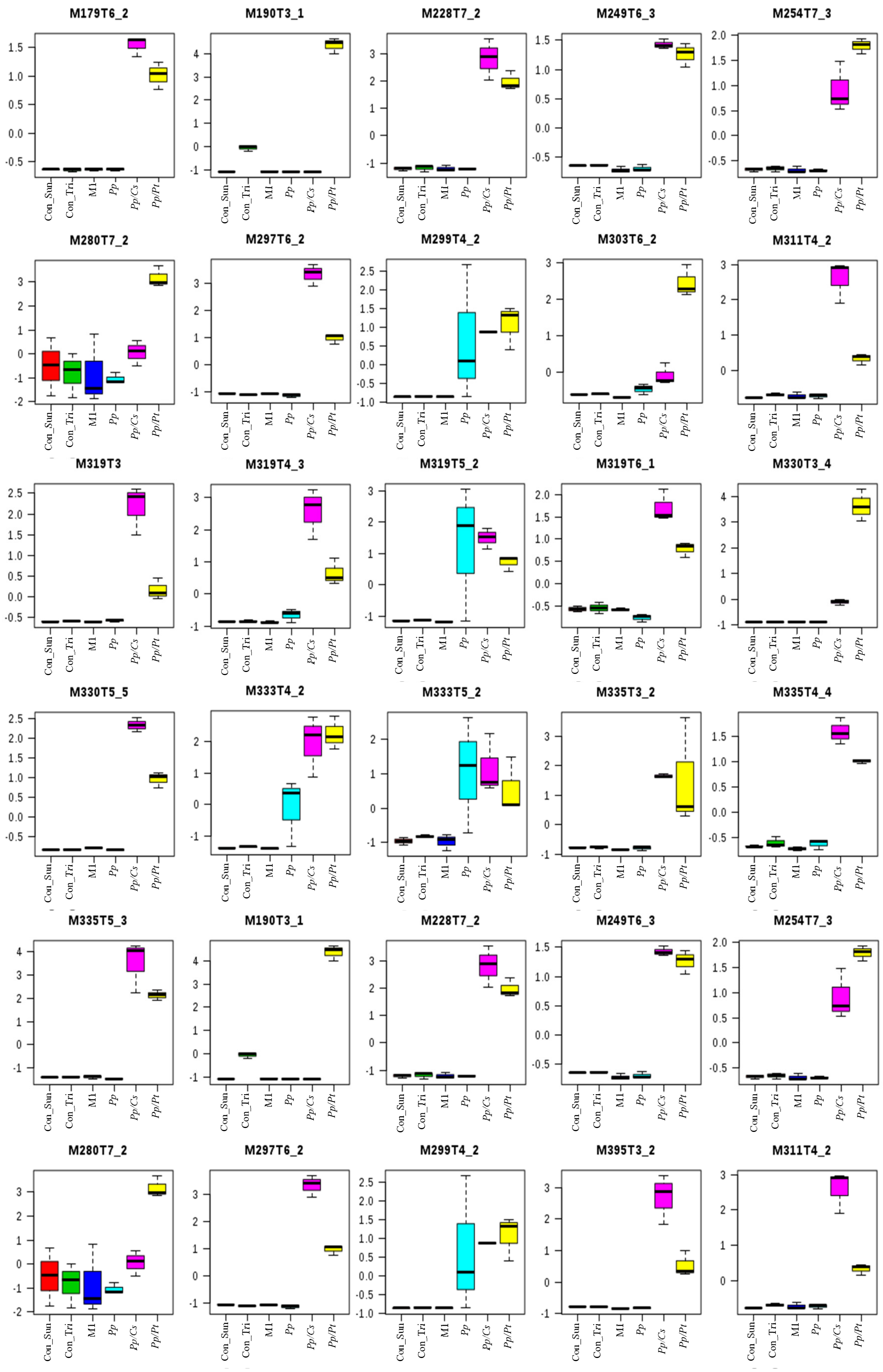

Figure S-2. Discriminant metabolites of *Pp*/*Pt* and *Pp*/*Cs* samples.

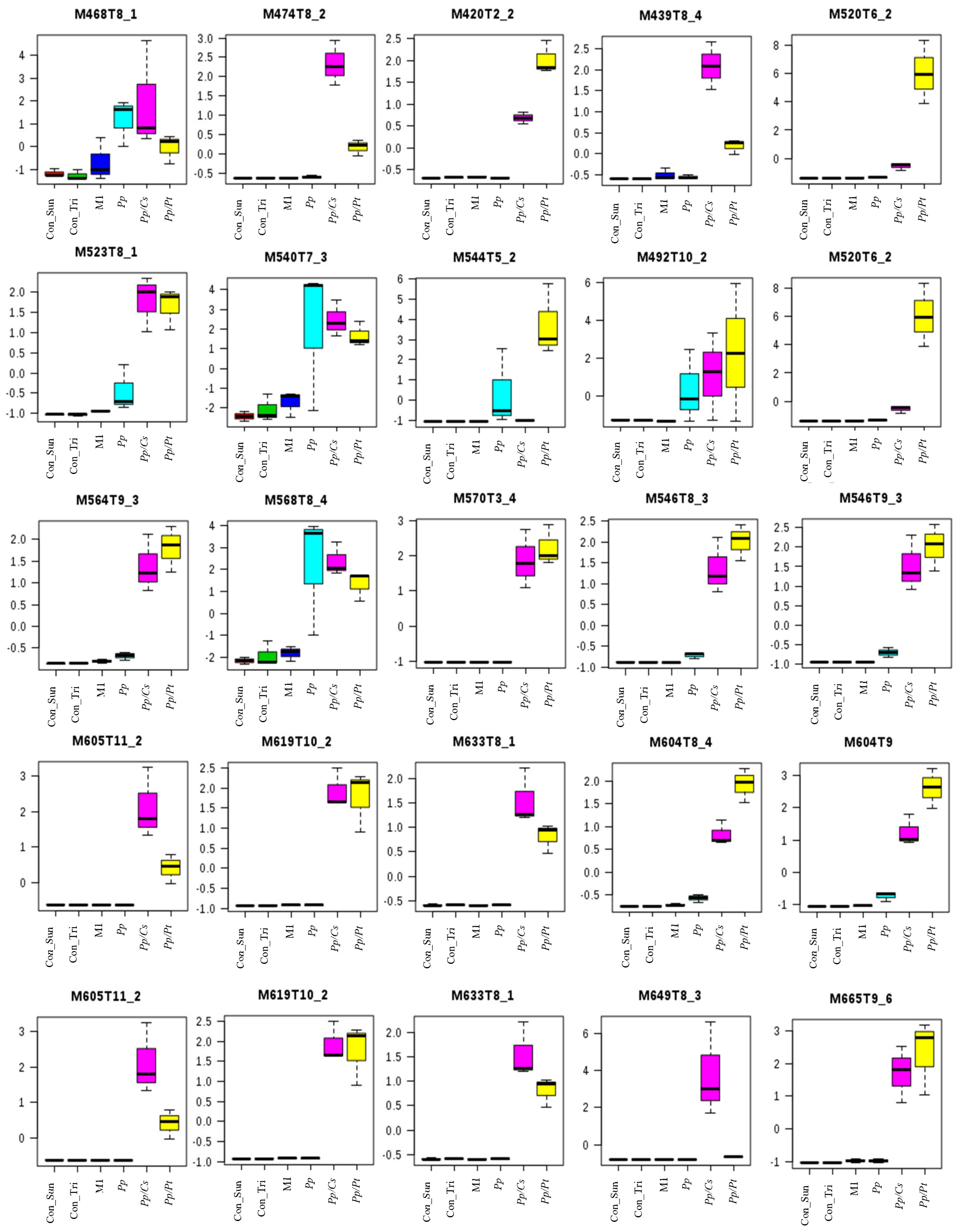

Figure S-3. Discriminant metabolites of *Pp*/*Pt* and *Pp*/*Cs* samples (continuation).

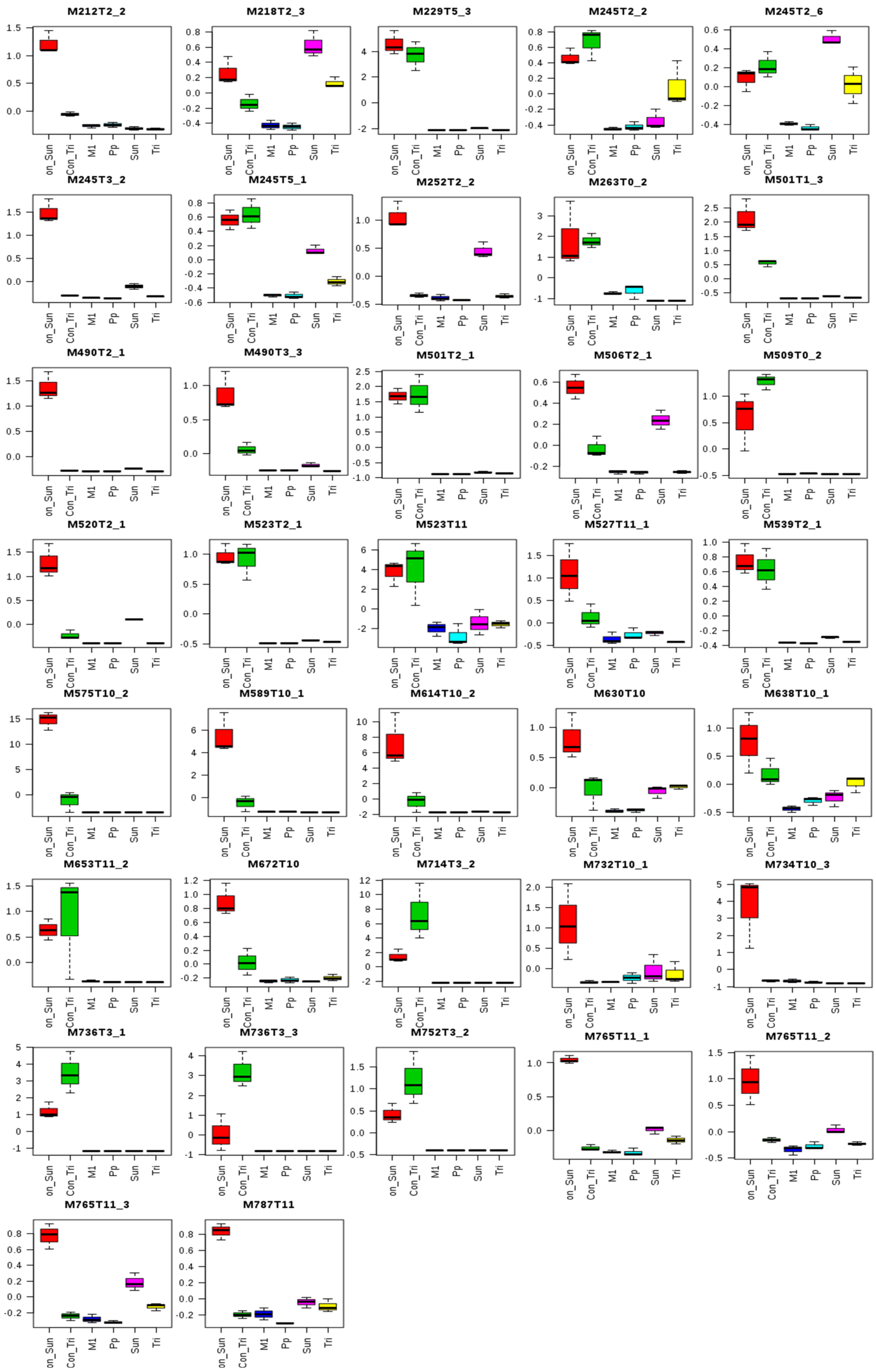
M212T2_2
M218T2_3
M229T5_3
M245T2_2
M245T2_6
M245T3_2
M245T5_1
M252T2_2
M263T0_2
M501T1_3
M490T2_1
M490T3_3
M501T2_1
M506T2_1
M509T0_2
M520T2_1
M523T2_1
M523T11
M527T11_1
M539T2_1
M575T10_2
M589T10_1
M614T10_2
M630T10
M638T10_1
M653T11_2
M672T10
M714T3_2
M732T10_1
M734T10_3
M736T3_1
M736T3_3
M752T3_2
M765T11_1
M765T11_2
M765T11_3
M787T11
on_Sun
Con_Tri
M1
Pp
Sun
Tri

Figure S-4. Discriminant metabolites of Con_*Pt* and Con_*Cs* samples. Sun=*Pt*/*Cs*; Tri=*Pp*/*Pt*.
